# Complexities of emotional responses to social and non-social affective stimuli in schizophrenia

**DOI:** 10.3389/fpsyg.2015.00320

**Published:** 2015-03-25

**Authors:** Joel S. Peterman, Esubalew Bekele, Dayi Bian, Nilanjan Sarkar, Sohee Park

**Affiliations:** ^1^Department of Psychology, Vanderbilt University, NashvilleTN, USA; ^2^Department of Electrical Engineering and Computer Science, Vanderbilt University, NashvilleTN, USA; ^3^Department of Mechanical Engineering, Vanderbilt UniversityNashville, TN, USA

**Keywords:** electromyogram, negative symptoms, positive symptoms, schizotypal personality, arousal, valence, self awareness, alexithymia

## Abstract

**Background**: Adaptive emotional responses are important in interpersonal relationships. We investigated self-reported emotional experience, physiological reactivity, and micro-facial expressivity in relation to the social nature of stimuli in individuals with schizophrenia (SZ).

**Method**: Galvanic skin response (GSR) and facial electromyography (fEMG) were recorded in medicated outpatients with SZ and demographically matched healthy controls (CO) while they viewed social and non-social images from the International Affective Pictures System. Participants rated the valence and arousal, and selected a label for experienced emotions. Symptom severity in the SZ and psychometric schizotypy in CO were assessed.

**Results**: The two groups did not differ in their labeling of the emotions evoked by the stimuli, but individuals with SZ were more positive in their valence ratings. Although self-reported arousal was similar in both groups, mean GSR was greater in SZ, suggesting differential awareness, or calibration of internal states. Both groups reported social images to be more arousing than non-social images but their physiological responses to non-social vs. social images were different. Self-reported arousal to neutral social images was correlated with positive symptoms in SZ. Negative symptoms in SZ and disorganized schizotypy in CO were associated with reduced mean fEMG. Greater corrugator mean fEMG activity for positive images in SZ indicates valence-incongruent facial expressions.

**Conclusion**: The patterns of emotional responses differed between the two groups. While both groups were in broad agreement in self-reported arousal and emotion labels, their mean GSR, and fEMG correlates of emotion diverged in relation to the social nature of the stimuli and clinical measures. Importantly, these results suggest disrupted self awareness of internal states in SZ and underscore the complexities of emotion processing in health and disease.

## Introduction

Emotional disturbances are core features of schizophrenia (SZ) and play a major role in functional outcome (Kring and Moran, 2008; Strauss and Herbener, 2011; Kring and Barch, 2014). Appropriate emotional responses within an ongoing social context are crucial to navigating interpersonal relationships but in individuals with SZ, components of emotional responses such as subjective experiences, expression, and physiological arousal may be fragmented rather than cohesively integrated. In general, subjective emotional experiences in-the-moment appear to be either intact or even exaggerated in SZ whether in the laboratory (Kring and Neale, 1996; Herbener et al., 2008; Cohen and Minor, 2010; Folley and Park, 2010; Cumming et al., 2011), or in daily life (Myin-Germeys and Delespaul, 2000), but the outward expression of emotions may be compromised. Facial expressions of emotion are reduced in SZ even when they report experiencing an emotion (Berenbaum and Oltmanns, 1992; Kring and Moran, 2008). Similarly, production of emotional prosody in speech has been shown to be impaired in SZ (Borod et al., 1989).

While overt facial expressions are reduced in SZ, recruitment of facial muscles involved in the production of emotional expressions may be intact, as detected by facial electromyography (fEMG; Kring et al., 1999). In general, increased zygomatic activity corresponds to positive/pleasant stimuli and increased corrugator activity to negative/aversive stimuli. fEMG data suggest that although overt facial expressions may be imperceptible to the observer, individuals with SZ may be engaging the facial muscles associated with these expressions, albeit at an attenuated level (Mattes et al., 1995; Wolf et al., 2006).

Emotional responses are accompanied by physiological changes that indicate valence and arousal (Levenson, 1992; Keltner and Gross, 1999; Cacioppo et al., 2000). Autonomic arousal in response to emotional stimuli can be assessed with the galvanic skin response (GSR) but the findings are mixed. GSR to emotional stimuli in SZ has been reported to be increased (Venables and Wing, 1962; Kring and Neale, 1996), reduced (Venables and Wing, 1962), or unchanged (Hempel et al., 2005, 2007). There are several possible reasons for these contradictory findings. The types of emotional stimuli used in these studies vary widely. Moreover, physiological indices of arousal are influenced by multiple factors, such as attentional orienting and the social significance or the salience of these stimuli, which in turn, are influenced by the social context or the environment.

Social context plays a central role in emotion processing in humans. Socially significant stimuli are known to enhance attentional orienting regardless of valence (Öhman et al., 2001; Schuller and Rossion, 2004), and the ability to orient to social stimuli is closely related to shared attention mechanism that facilitates social interactions (Dawson et al., 1998; Langton and Bruce, 1999). SZ is characterized by marked impairments in social functioning from the premorbid stage and throughout the course of the illness (Bellack et al., 2007; Green et al., 2008; Couture et al., 2011). Similar to autism, individuals with SZ show abnormal responses to social compared with non-social stimuli. For example, both groups show abnormal attentional orienting to social stimuli such as faces (Sasson et al., 2007), and show impairments in perception of movements elicited by living things (i.e., social stimuli; Blake et al., 2003; Kim et al., 2005, 2011, 2013; Freitag et al., 2008; Klin et al., 2009; Peterman et al., 2014).

Anomalous self awareness of one’s internal states (e.g., Sass and Parnas, 2003; Lysaker and Dimaggio, 2014) including impaired interoception (Postmes et al., 2014) could also contribute to the discrepancy between physiological arousal data and the self-reported emotional experience in individuals with SZ, leading to an inability to consciously identify, and describe one’s emotional experience, known as alexithymia (Lane et al., 1997). Thus, individual differences in one’s ability to match internal states to verbal categories (e.g., alexithymia), and associated impairments in self awareness also play a role in subjective experiences of emotions. Alexithymia has been reported in SZ (Van’t Wout et al., 2007), and schizotypy (Seghers et al., 2011; Aaron et al., 2015) and may be related to ‘somatic aphasia,’ which refers to impaired identification and recognition of one’s own somatic states (Gao et al., 2012). Difficulties in identification, categorization, and understanding of internal states may be driven by meta-cognitive deficits (see Lysaker et al., 2011, 2013; Lysaker and Dimaggio, 2014) in addition to multiple problems at the sensory input stage of information processing (e.g., Doniger et al., 2001; Leitman et al., 2010; Postmes et al., 2014). Thus, an interaction of faulty meta-cognitive processes and impaired perceptual input may both contribute to anomalous emotional experiences in SZ.

Social context contributes to top–down meta-cognitive processes that play an important role in our daily emotional experiences. Social and emotional processes are intertwined throughout the nervous system at all levels of information processing (Norris et al., 2004). Although, emotion theories tend to emphasize valence and arousal dimensions or specific basic categories of emotions, past studies have also shown that sociality plays a very important role in processing emotional valence (Britton et al., 2006a,b; Harris et al., 2007), and more generally, categorization (Wood et al., 2003) as well as general information processing (Gilbert et al., 2007). Influence of social nature of stimuli emotions is largely based on the presence of human (or living) forms, and whether one is sensitive to the social significance of these stimuli has implications for psychiatric conditions. For example, in autism literature, the distinction between social and non-social stimuli is clearly articulated and the difference between individuals with autism and neurotypical participants emerges in their differential responses to social and non-social stimuli. Social stimuli show processing advantages compared to non-social stimuli in healthy individuals, but in individuals with autism, such advantages are abolished and there may even be a processing benefit for non-social stimuli (e.g., Dawson et al., 1998; Sasson et al., 2008; South et al., 2008; Di Martino et al., 2009; Baranek et al., 2013). In the present manuscript, we focus on the effects of the social nature of stimuli on emotion experience, and whether social and non-social stimuli evoke different emotional experiences in SZ versus control participants.

In SZ, differential physiological responses to social and non-social stimuli have been observed using the post-auricular reflex paradigm (Aaron et al., 2013), which indexes automatic response to pleasant stimuli (Benning et al., 2004); the postauricular reflex was enhanced for positively valenced social scenes from the International Affective Pictures System (IAPS; Lang and Bradley, 2007; Lang et al., 2008; e.g., erotica and nurturing scenes containing humans), but not for pleasant non-social pictures (e.g., beautiful nature). Importantly, if the sociality factor had not been examined, the authors would have concluded that the physiological responses to pleasant emotional stimuli were reduced overall in SZ, missing this crucial interaction. These findings underscore the importance of examining sociality dimension in emotion tasks.

In the current study, we examined subjective emotional experiences, physiological arousal, and facial electromyographical response to visual stimuli in relation to the social nature of these images. Social stimuli were selected from the IAPS (Lang et al., 2008) across three valence categories (positive, neutral, and negative). A non-social set of images matched in valence and arousal ratings to social stimuli (Lang et al., 2008) were also selected to compare emotional responses across socio-emotional dimensions. We assessed subjective experiences via self-reported ratings, emotional expressivity with fEMG, and physiological arousal with skin conductance. Given past findings (Berenbaum and Oltmanns, 1992; Kring and Neale, 1996; Doop and Park, 2006), we expected the relationship between physiological measures and subjective ratings of emotional experiences would be anomalous in SZ, given their reduced self awareness and impaired ability to accurately describe and explain internal states. With respect to expressivity measured by fEMG, we expected individuals with SZ to show reduced activity of the zygomatic and corrugator muscles. The findings in the physiology of emotional responses in SZ are mixed but we decided to examine GSR to social and non-social stimuli to test whether physiological responses to social stimuli would be abnormal in SZ.

## Materials and Methods

### Participants

Twelve medicated outpatients with SZ were recruited from private care facilities in Nashville, TN, USA. Diagnoses were confirmed with the structured clinical interview for DSM-IV (SCID; First et al., 2002). Symptoms were assessed using the Scale for the Assessment Positive Symptoms (SAPS; Andreasen, 1984b) and the Scale for the Assessment Negative Symptoms (SANS; Andreasen, 1984a).

Twelve healthy controls were recruited from the same community via advertisements. They were screened for current and prior history of Axis I disorders using the SCID (First et al., 2002) and a history of psychosis in their first-degree relatives.

Exclusion criteria for both groups were as follows: intelligence (IQ) < 85, a prior history of head injury or neurological disorder or history of drug use in the year prior to the study. Premorbid IQ was estimated using the North American Adult Reading Test (NAART; Uttl, 2002). Current social functioning was assessed with the Social Functioning Scale, a semi-structure interview (Birchwood et al., 1990). Schizotypy in CO was assessed using the Schizotypal Personality Questionnaire (Raine, 1991). All participants had normal or corrected-to-normal vision. Participants gave written informed consent as approved by the Vanderbilt Institutional Review Board. The two groups were matched on age, gender, estimated IQ, but not education. However, SZ had at least high school education. (see **Table 1** for demographic, clinical, and medication information).

**Table 1:** Demographic and clinical information.

	Schizophrenia (SZ)	Controls (CO)	Statistical test	*p*-value
	Mean (SD)	Mean (SD)		
Age	44.9 (9.9)	45.7 (9.4)	*t* = -0.21	0.83
Gender	6 F/6 M	4 F/8 M	chi^2^ = 0.68	0.40
Years of education	13.4 (2.1)	15.4 (2.2)	*t* = -2.2	0.03
IQ	107.1 (6.8)	106.4 (7.4)	*t* = 0.22	0.82
Years of illness	23.55 (9.7)	N/A	N/A	N/A
SAPS	12.9 (7.7)	N/A	N/A	N/A
SANS	24.5 (11.4)	N/A	N/A	N/A
SPQ	N/A		N/A	N/A
Positive		2.75 (4.1)		
Negative		4.00 (3.8)		
Disorganized		2.58 (3.8)		
CPZ	359.63 (247.6)	N/A	N/A	N/A

*IQ, intelligence quotient estimated by North American Adult Reading Test (NAART); SAPS, Scale for the Assessment of Positive Symptoms; SANS, Scale for the Assessment of Negative Symptoms; SPQ, Schizotypal Personality Questionnaire (Raine, 1991); CPZ, Chlorpromazine equivalent dose (mg/day); N/A, not applicable.*

### Stimuli and Equipment

The stimuli were selected from the IAPS (Lang et al., 2008). To assess the effect of sociality on emotional responses, we selected social and non-social images from the positive, negative, and neutral images to create six categories of image blocks^.^ Social images always contained one or more people. Due to previous research indicating sex differences in self-reported valence and arousal ratings of some IAPS images, the stimulus sets for male and female participants differed slightly (Lang and Bradley, 2007). The selected IAPS images were as follows. For male participants: 1274, 2200, 4599, 4601, 4651, 4700, 6212, 6250, 6510, 6560, 7006, 7140, 7190, 7235, 7270, 7350, 7460, 7480, 7550, 7620, 9140, 9210, 9290, 9300. For female participants: 1274, 2200, 4599, 4601, 4640, 4700, 6212, 6250, 6560, 7006, 7140, 7190, 7235, 7270, 7350, 7460, 7480, 7550, 9140, 9210, 9290, 9373.

Importantly, across these two sets of stimuli for men and women, the average valence and arousal ratings, based on those provided in the IAPS database did not significantly differ for any of the image categories. Furthermore, the average valence and arousal ratings did not significantly differ between social and non-social categories.

**FIGURE 1:**
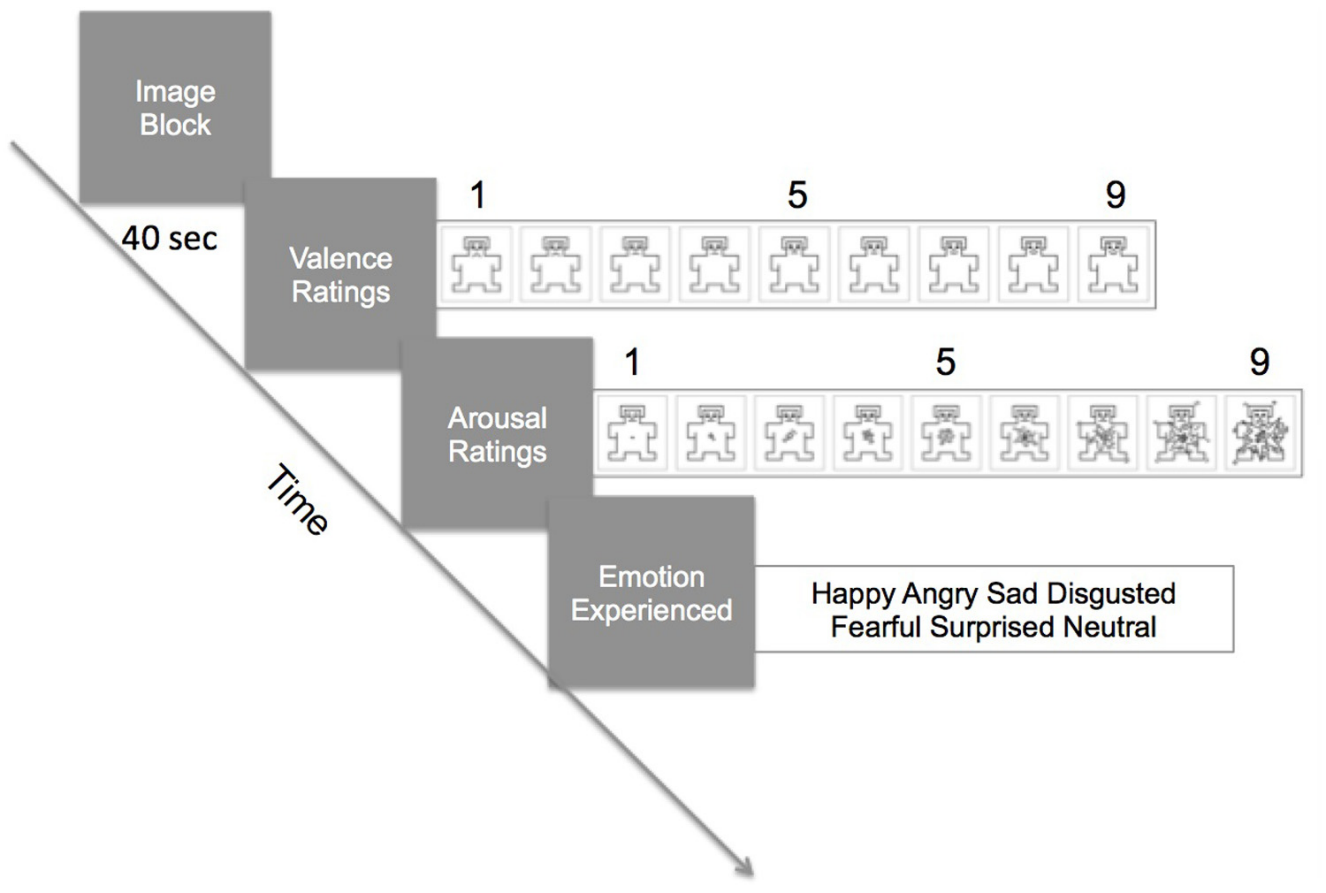
**Procedure of the task**. In a trial, participants viewed a block of four images for 40 s (10 s per image). Galvanic skin response (GSR) and facial electromyography (fEMG) were continuously recorded during the viewing. Immediately after the block, they rated the valence and the arousal level using the Self-Assessment Mannikin Scale (SAM). Then, they selected from a choice of six words which emotion they felt during viewing of the images.

Stimuli were presented on a 24” monitor (1024 × 768 resolution) 70–80 cm from the participant with Unity3D software (Unity Technologies, San Francisco, CA, USA). Stimuli were shown in category blocks of four images, at 10 s per image (40 s per block). Immediately after a block, participants were asked to indicate, with a mouse, their valence and arousal ratings using the *Self-Assessment Mannikin Scale* (SAM; Lang, 1980). The far left SAM indicated very negative or very unarousing and the far right SAM indicated very positive/pleasant or very arousing (see **Figure 1**). Then, participants selected the emotion they were experiencing out of six choices (happy, angry, afraid, sad, disgusted, neutral). There were six blocks, one for each category (social positive, social negative, social neutral, non-social positive, non-social negative, non-social neutral). A block design was used to maximize the experience of one category type. The order of presentation of blocks was randomized.

### Physiological Monitoring and Processing

A wireless Bionomadix (BIOPAC Systems Inc., Goleta, CA, USA) physiological monitoring system was used to record three channels, sampled at 1000 Hz: GSR (μS ) and two electromyograms (mV; EMG; corrugator supercilli and zygomaticus major). Physiological signals were post-processed for analysis. After filtering to reject outliers and artifacts, they were standardized (Mean = 0, SD = 1).

### Procedure

Wireless sensors were placed on the participant’s face and non-dominant hand. The sensors measuring corrugator activity were placed above their left eyebrow with one of the sensors aligning to the pupil when looking straightforward and above the tear duct. The sensors measuring zygomatic activity were placed about a third of the distance from the left-side corner of the mouth and the lower crease where the ear attaches to the head. The GSR sensors were placed on the index and ring fingertips of the hand.

After all the sensors were placed and checked for signal strength, participants sat for 3 min quietly to allow for settling of physiological responses and collection of baseline responses. After the collection of baseline data, participants performed the IAPS viewing task. GSR and fEMG were recorded during the image blocks. Each trial began with participants viewing a block of four stimuli sequentially (10 s per picture). These four images belonged to the same category (i.e., social positive, social negative, social neutral, non-social positive, non-social negative, or non-social neutral).

Immediately after the presentation of the block, subjects indicated how positive or negative the image block made them feel (valence rating), and the degree of arousal (arousal rating) with the SAM using a mouse. After these ratings, they indicated which emotion they felt by selecting from six words on the computer screen with the mouse. (see **Figure 1**). Then, the next image block was presented and the task repeated until all six categories of image blocks were rated.

### Data Analysis

#### Arousal Data

The GSR data were first smoothed with a median filter for outlier removal and down sampled by a factor of 10. The threshold of amplitude of GSR was 0.05 microsiemens. We employed a bandpass filter and a notch filter to remove interference at 60 Hz. The tonic and phasic responses were separated, based on their frequencies. First, the phasic response was extracted using high frequency bandpass and the phasic response was subtracted from the original GSR signal to obtain the slow moving tonic response baseline. The tonic response refers to the slow moving background (baseline) component of the signal, which is produced regardless of external stimuli presentation, whereas phasic response refers to the high frequency signal that rides on top of the tonic baseline and may be produced in response to external stimuli in the case of event-related skin conductance response.

#### Electromyography

First a local optimized median filter was used to remove outliers from the EMG data. Then the data were processed with a bandpass filter and notch filter to remove noise. Two sets of features were extracted after preprocessing of the data: frequency features containing median, mean, SD which refer to the intensity of muscle activity and burst features that provide the counts of muscle activities per unit of time.

To assess group differences in self-reported valence and arousal, 2 × 2 × 3 mixed repeated-measure ANOVAs were conducted for both variables with group (SZ, CO) as the between-group variable and sociality of images (Social, Non-Social) and valence of the image (Positive, Negative, Neutral) as the within-variables. Specifically, we were interested in the following interactions: group-by-valence, sociality-by-valence, and group-by-sociality-by-valence. To test group differences in self-reported emotion label, chi-square analyses were conducted for the six category blocks and the participants’ choice of emotion words.

To assess differences in physiological response to the social and non-social images, a repeated-measures multivariate analysis of variance (MANOVA) was conducted on the percent change from baseline for the GSR. To assess differences in facial expressivity response to the social and non-social images, repeated-measures MANOVAs were conducted on the % change in activity from baseline for the fEMG recordings.

All analyses are two-tailed unless otherwise specified and trends are reported if *p* < 0.10. Effect sizes are reported for all significant and trend analyses with partial eta-squared.

## Results

### Self-Report Findings

#### Self-Reported Valence

Self-Assessment Mannikin Scale ratings were converted a numerical scale ranging from 1 (far left SAM) to 9 (far right SAM). There was a main effect of group [*F*(1,22) = 4.5; *p* = 0.05; $\eta_{p}^{2}$ = 0.17]. Overall, SZ rated the images as more pleasant (positive) than CO. The main effect of valence was significant [*F*(2,44) = 56.30; *p* < 0.0001; $\eta_{p}^{2}$ = 0.72]. Both groups rated positive images as more pleasant (SZ: 8.1; CO: 7.2) than the neutral (SZ: 6.4; CO: 5.2) and negative images (SZ: 3.5; CO: 2.6). The group-by-valence interaction [*F*(2,44) = 0.054; *p* = 0.95], and sociality-by-valence interaction [*F*(2,44) = 0.65; *p* = 0.52] were not significant. Valence ratings for the social and non-social images did not differ across groups. Finally, the group-by-sociality-by-valence interaction was not significant [*F*(2,44) = 0.59; *p* = 0.56].

#### Self-Reported Arousal

**FIGURE 2:**
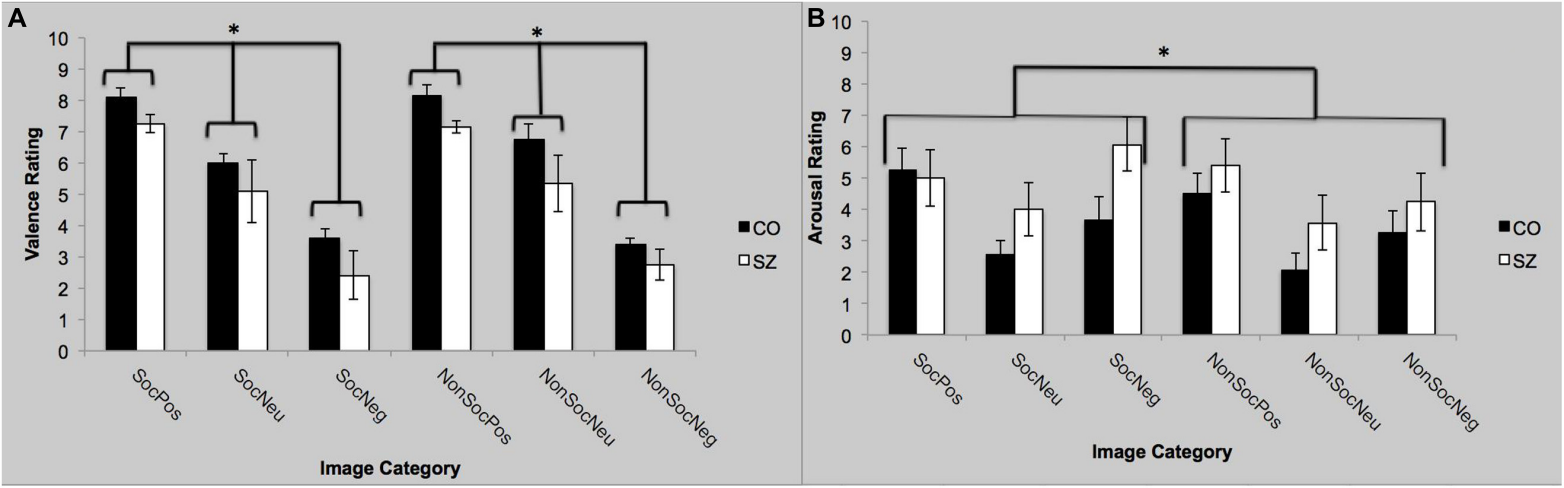
**Self-reported valence and arousal ratings with SAM. (A)** Both groups rated the valence to be significantly more positive (pleasant) for the positive images compared to the neutral images and the negative images to be more significantly more negative than the neutral images. **(B)** Both groups rated the positive and negative images to be more arousing than the neutral images. Furthermore, the social images were rated as more arousing than the non-social images, regardless of valence. SocPos, social positive; SocNeu, social neutral; SocNeg, social negative; NonSocPos, non-social positive; NonSocNeu, non-social neutral; NonSocNeg, non-social negative. ^∗^*p* < 0.05.

Self-Assessment Mannikin Scale ratings were converted a numerical scale ranging from 1 (far left SAM) to 9 (far right SAM). There was no main effect of group [*F*(1,22) = 2.29; *p* = 0.14], but there was a main effect of sociality on the arousal ratings [*F*(1,22) = 4.39; *p* = 0.05; $\eta_{p}^{2}$ = 0.17]. Regardless of valence, the social image blocks were rated as more arousing than the non-social images. The main effect of valence was significant [*F*(2,44) = 7.60; *p* = 0.001; $\eta_{p}^{2}$ = 0.26]. The positive and negative images were rated as more arousing than the neutral images, but they did not differ from each other. The group-by-valence interaction was not significant [*F*(2,44) = 1.02; *p* = 0.37]. The sociality-by-valence interaction was not significant [*F*(2,44) = 1.04; *p* = 0.36]; the presence of social context did not appear to have an effect on valence-specific ratings of arousal, across both groups. Finally, the group-by-sociality-by-valence interaction was not significant [*F*(2,44) = 1.83; *p* = 0.17]. See **Figure 2**.

#### Self-Report of Experienced Emotion

There were no significant group differences for the self-reported emotional experience (i.e., happy, angry, afraid, sad, disgusted, neutral) across all social and non-social IAPS image categories. (χ^2^ = 1.4–4.5; *p* > 0.20). The two groups were in agreement with respect to labeling the emotions evoked by IAPS stimuli.

### Physiological Reactivity

#### Tonic Galvanic Skin Response

There was a main effect of group; SZ showed a stronger GSR overall compared to CO [*F*(3,42) = 2.82; *p* = 0.05; $\eta_{p}^{2}$ = 0.17], which is interesting since the two groups did not differ in their self-reported arousal (see above). There was no main effect of sociality; both groups showed similar GSR to social and non-social images [*F*(3,42) = 0.68; *p* = 0.57]. Finally there was no group-by-sociality interaction [*F*(3,42) = 0.36; *p* = 0.78].

#### Galvanic Skin Response Rates

There was no main effect of group [*F*(3,42) = 0.59; *p* = 0.62]. There was no main effect of sociality [*F*(3,42) = 0.28; *p* = 0.84]. However, there was a significant group-by-sociality interaction [*F*(3,42) = 2.88; *p* = 0.05; $\eta_{p}^{2}$ = 0.17]. *Post hoc* analyses indicate that for neutral pictures, the two groups did not differ on the mean GSR rates to social stimuli [*t*(22) = -0.66; *p* = 0.52] but for non-social pictures, physiological arousal was significantly elevated in SZ compared to CO [*t*(22) = 2.31; *p* = 0.03]. Thus, abnormally high arousal to non-social neutral stimuli in SZ is driving the group-by-sociality interaction.

### EMG Analyses

#### Zygomaticus Major

There was a trend for a main effect of group [*F*(3,42) = 2.45; *p* = 0.08; $\eta_{p}^{2}$ = 0.15]. *Post hoc* analyses indicate there was a trend for SZ to show greater mean activity of the zygomatic muscle during viewing of positive images compared to CO [*F*(1,44) = 3.49; *p* = 0.07]. There was no main effect of sociality [*F*(3,42) = 0.34; *p* = 0.80]. The group-by-sociality interaction was not significant [*F*(3,42) = 0.08; *p* = 0.97].

#### Corrugator

**FIGURE 3:**
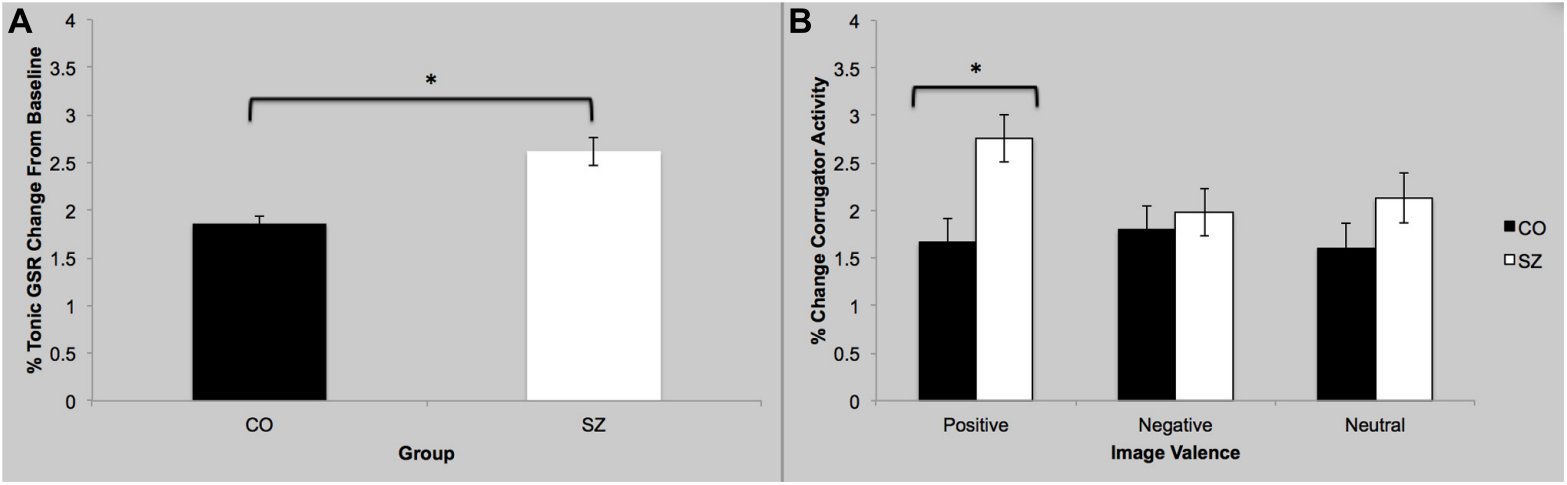
**Galvanic skin response and facial EMG results. (A)** Overall, SZ showed greater GSR response while viewing images compared to CO. **(B)** SZ showed greater activity of the corrugator supercilli muscle while viewing the positive images compared to CO. ^∗^*p* < 0.05.

There was a main effect of group [*F*(3,42) = 3.99; *p* = 0.01; $\eta_{p}^{2}$ = 0.22], see **Figure 3**. Specifically, SZ showed greater mean corrugator response during viewing of positive images compared to CO [*F*(1,44) = 9.59; *p* = 0.003; $\eta_{p}^{2}$ = 0.18]. There was no main effect of sociality [*F*(3,42) = 0.23; *p* = 0.88]. There was no group-by-sociality interaction [*F*(3,42) = 0.13; *p* = 0.94].

### Correlations with Clinical Variables

There were no significant correlations among the self-report ratings, arousal, and fEMG across both groups. We report within-group associations below.

#### Schizophrenia

Self-reported arousal during viewing of neutral social images was positively correlated with SAPS (ρ = 0.79; *p* = 0.002). Thus, increased positive symptoms severity was associated with greater arousal for neutral social stimuli. SANS was negatively associated with the mean EMG activity in the corrugator during viewing of negative social images (ρ = -0.62; *p* = 0.03), and the mean EMG activity in the zygomatic muscle during viewing of positive non-social images (ρ = -0.71; *p* = 0.01).

#### Controls

The disorganized subscale of the SPQ was positively associated with arousal ratings during viewing the neutral social images (ρ = 0.60; *p* = 0.04); those with elevated schizotypal traits tended reported greater arousal while viewing neutral social images. The disorganized subscale of the SPQ was negatively associated with self-reported valence ratings for the negative social images (ρ = -0.80; *p* = 0.002) and the negative non-social images (ρ = -0.70; *p* = 0.003); those with elevated schizotypal traits tended to give lower ratings for negative images.

## Discussion

We conducted a comprehensive assessment of the emotional responses to visual images in SZ and matched CO, by collecting self-reported valence and arousal ratings, as well as measuring physiological arousal and fEMG.

Labeling of emotion experience seems to be intact in SZ, as both groups generally indicated experiencing the same emotions when asked to select a word that matched what they felt upon viewing IAPS stimuli. However, it must be noted that they were choosing from a set of six words rather than being asked to describe their feelings. Therefore, we are not able to determine whether there are subtle difficulties in labeling emotions.

With respect to valence, SZ rated stimuli to be more pleasant than CO, which replicates previous findings of elevated positive ratings of affective stimuli (Doop and Park, 2006; Folley and Park, 2010; Cumming et al., 2011), and not inconsistent with findings of intact pleasantness ratings of IAPS stimuli (Heerey and Gold, 2007; Herbener et al., 2007). Previous research has indicated that SZ and CO report experiencing similar levels of emotion when presented with affective stimuli (Kring and Neale, 1996; Myin-Germeys and Delespaul, 2000). In our study, both groups reported experiencing similar levels of arousal, but one physiological index of arousal, the GSR, told another story. GSR was increased in SZ, which perhaps hints at altered awareness or calibration of internal states; they seem to require greater levels of arousal than the healthy participants to reach the same conscious decision.

Emotional awareness may depend on integrating sensorimotor and interoceptive information with an interpretation of the external situation in real time (Terasawa et al., 2013). The role of bodily sensations and interoceptive awareness in emotion experience is unclear but interoception and somatosensory processing are compromised in the SZ-spectrum (Peled et al., 2003; Chang and Lenzenweger, 2005; Linnman et al., 2013) and may contribute to alexithymia (Van’t Wout et al., 2007; Aaron et al., 2015). Although very little is known about the etiology of alexithymia in SZ, it is possible that there is an abnormal integration of internal signals with conscious, categorical processing in the brain. Future research should further elucidate the dysconnectivity between self-reported emotions and internal states in SZ.

For both groups, self-reported arousal was influenced by the presence of social components in stimuli. This finding was unexpected given that we had carefully matched for normative valence and arousal ratings (Lang et al., 2008) across the social and non-social images. Nonetheless, participants reported that the social images (i.e., containing humans) were more arousing than non-social images. This finding underscores the importance of social context in emotional experience (Keltner and Haidt, 1999). Moreover, in terms of physiological arousal, there was a curious interaction between group and sociality on the mean GSR rate. The GSR rates of the two groups do not differ for the social neutral images but for non-social images, it was elevated for SZ compared to CO. One possibility is that the social and non-social boundary may be blurred in SZ (see Kim et al., 2011). Inanimate objects are considered non-social but there are conditions under which they acquire animistic and social qualities, notably during hallucinations and after prolonged social isolation (see Hoffman, 2007). Hoffman has proposed that isolation leads to a state of ‘social deafferentation’ (analogous to physical amputation), which gives rise to social delusions and hallucinations and thus, forms a basis for psychotic experiences in SZ. It might be important to re-examine our conceptualization of ‘social vs. non-social’ categories in the context of psychosis since what may be non-social to healthy participants may be alive with meaning in those with SZ.

With respect to fEMG, interesting differences emerged between SZ and CO. While the finding of reduced *overt* facial expressions in SZ is relatively consistent (Kring and Moran, 2008), studies analyzing the muscular activity of the face has been more variable (Mattes et al., 1995; Kring et al., 1999;Wolf et al., 2006). In our study, the activity of the corrugator muscle in SZ was significantly greater than that in CO, especially for positive images. Since corrugator muscles are implicated in the expression of negative emotions (e.g., frown), the fact that pleasant images increase corrugator activity is counterintuitive. However, mixed emotions do exist and increase with age (see Carstensen et al., 2000; Ong and Bergeman, 2004). Although SZ and CO did not differ significantly in their self-reported emotions, their responses were artificially constrained by the task requirement (i.e., selection of one out of six emotion words). Therefore, it is not possible to determine whether the experienced emotions were ‘pure,’ undifferentiated or mixed. In this context, the insightful observation that in persons with SZ, positive and neutral stimuli may “co-activate hedonic and aversive emotions” seems particularly relevant (Cohen and Minor, 2010).

Pleasant, positive images elicited somewhat greater zygomatic activity in SZ than CO. Given the previous findings of reduced overt facial expression in SZ compared with CO (see Kring and Moran, 2008), it is puzzling as to why the activity of the muscles underlying overt facial expressions is enhanced in SZ in our study. SZ did not differ from CO in their choice of labels for the emotions experienced so at least forced-choice labeling emotions seems to be intact. However, our fEMG data suggest that embodied simulation of those emotions and/or the expression of these emotions may be dissociated from the subjective, experiential component.

Within the patient group, fEMG activity was negatively associated with the severity of negative symptoms; reduced fEMG in those with elevated negative symptoms may be manifested as flat affect. In CO, disorganized schizotypy was associated with increased arousal to neutral social images, which seems maladaptive; since neutral scenes are supposed to lack emotional content (e.g., a person standing under an umbrella), they should be least arousing compared with positive and negative stimuli.

With respect to the physiological indices of emotion experience, the skin conductance data indicated areas of difference between SZ and CO. Overall, SZ showed greater tonic GSR than CO. Heightened physiological arousal has been reported in a subset of SZ who are more socially withdrawn (Venables and Wing, 1962). In our study, SZ reported lower social engagement and more time spent alone than CO, as indicated by the Social Functioning Scale (Birchwood et al., 1990). Thus, increased physiological arousal may have been driven by social isolation in SZ.

Lastly, we turn to the potential role of the social context in emotion processing in SZ. Although self-reported arousal was increased for social images for both groups, physiological responses to the non-social images diverged. In SZ, physiological arousal was elevated for non-social images compared with CO. Given the mundane and non-emotional content of the non-social images, greater arousal in SZ suggests over-interpretation of external stimuli that could eventually lead to delusions, and/or mis-calibration of internal states. Interestingly, self-reported arousal ratings for the social neutral images were significantly related to positive symptoms in SZ. More psychotic patients found neutral social scenes more arousing. The very boring nature of the social neutral images (e.g., man sitting at a computer, a person standing under an umbrella) render certain ambiguity, which may then trigger greater elaboration and interpretation of these scenes. This may partly explain increased threat sensitivity in SZ (Henry et al., 2010). A similar pattern was also observed in CO. Elevated SPQ-disorganized syndrome and arousal ratings for the social neutral images were correlated. These results imply that so-called ‘neutral’ images are not necessarily emotion-less and we must carefully consider individual differences in the top–down processing of standardized emotional stimuli. Future studies should investigate the process of interpretations that contribute to emotional experience when presented with ambiguous stimuli.

There are limitations to our study. The sample size was small but this study is one of the very few to examine subjective emotional experiences, physiological index of arousal (skin conductance), and fEMG within the same experiment in demographicaly matched SZ and CO. However, future studies with larger samples are warranted. Another limitation is the use of static images for evoking emotional responses. While IAPS stimuli have shown to be sufficient in a wide range of emotion studies (see Bradley et al., 2001), their ecological validity is circumscribed. We live in a dynamic and ever-changing world, which engages our emotions in a similarly dynamic way. A shift toward dynamic stimuli will allow for the assessment of emotional responses that are closer to actual everyday experiences and simulate social interactions, an approach that has been successfully used in autism intervention (Bekele et al., 2013).

In summary, we found pockets of intact and anomalous indices of emotional processes in SZ, with dissociation of self-reported emotions from the physiological and expressive indices emotion. Such splitting of subjective feelings from internal signals perhaps reflects what Eugen Bleuler meant by SZ, and points to the significance of interventions targeting metacognitive skills as well as psychophysiological and sensory abnormalities (Lysaker and Dimaggio, 2014).

## Conflict of Interest Statement

The authors declare that the research was conducted in the absence of any commercial or financial relationships that could be construed as a potential conflict of interest.
